# Treatment of irreducible intertrochanteric femoral fracture with a minimally invasive clamp reduction technique via the anterior approach

**DOI:** 10.1186/s13018-023-03641-8

**Published:** 2023-03-04

**Authors:** Jinya Qiu, Zhen Jiang, Liang Han, Xingwei Li, Rui Zhang, Bin Wu, Fenghua Zhu, Yifeng Zhao

**Affiliations:** 1 Department of Clinical Medicine, Jining Medical University, 133 Hehua Road, Taibai Lake New District, Jining, 272067 Shandong People’s Republic of China; 2 Department of Traumatic Orthopedics, Affiliated Hospital of Jining Medical University, 129 Hehua Road, Taibai Lake New District, Jining, 272007 Shandong People’s Republic of China; 3 Department of Orthopedics, Wenshang People’s Hospital, 1, Dehui Road, Wenshang County, 272501 Shandong People’s Republic of China

**Keywords:** Irreducible femoral intertrochanteric fracture, Anterior approach, Clamp reduction, Minimally invasive technique

## Abstract

**Objective:**

To investigate the efficacy of the minimally invasive clamp reduction technique via the anterior approach in the treatment of irreducible intertrochanteric femoral fractures.

**Methods:**

From January 2015 to January 2021, 115 patients (48 males and 67 females) with irreducible intertrochanteric femoral fractures were treated. The average age of the patients was 78.7 (45–100 years old). The types of injuries were falls (91 cases), traffic accidents (12 cases), smashing (6 cases), and high falling (6 cases). The duration between injury and surgery ranged from 1 to 14 days, with an average of 3.9 days. The distribution of AO classification was as follows: 31-A1 in 15 cases, type 31-A2 in 67 cases and 31-A3 in 33 cases.

**Results:**

All patients achieved good reduction, with fracture reduction times ranging from 10 to 32 min (mean of 18 min), and were followed up for 12–27 months after surgery (mean of 17.9 months). Two patients with pronation displacement of the proximal fracture segment died of infection or hypostatic pneumonia after internal fixation failure; one patient with failed internal fixation switched to joint replacement. After internal fixation, the lateral wall of six reversed intertrochanteric femoral fractures showed repronation and abduction displacement, but all fractures achieved bony healing. The rest of the patients did not lose fracture reduction, and all fractures achieved bony healing with a healing time ranging from 3 to 9 months (mean of 5.7 months). While two patients died and one patient exhibited failed internal fixation and thus switched to joint replacement, 91 of the remaining 112 patients had an excellent Harris score of the hip joint function at the final follow-up, while 21 patients had a good Harris score.

**Conclusion:**

The minimally invasive clamp reduction technique via the anterior approach for the treatment of irreducible intertrochanteric femoral fractures is simple, effective and minimally invasive. In the case of irreducible intertrochanteric femoral fractures associated with lateral wall displacement, the lateral wall needs to be strengthened after clamp reduction and intramedullary nail fixation to avoid loss of reduction and failure of internal fixation.

Intertrochanteric femoral fractures are common in traumatic orthopedics, and they mostly occur among elderly individuals. Traction bed closed reduction and intramedullary nail internal fixation have become the main treatment modalities [1]. Although the majority of fractures can be successfully reduced after abduction, external rotation, adduction and internal rotation of the hip with traction bed traction [2, 3], a small proportion of closed reductions fail and require limited incision and auxiliary reduction [4–8]. These fractures are called “irreducible intertrochanteric femoral fractures" and account for 3–17% of all intertrochanteric fractures [6]. There are many clinical reduction approaches, such as pulling the distal fracture segment with a bone hook, clamping the fracture segment with reduction forceps [9], prying the cephalocervical fracture segment backward through the enlarged spiral blade entrance with the periosteal stripper [10], cutting the strangulated iliac muscle [11, 12], and jacking the distal fracture segment with the bucking bar[13–18]. Yingze Zhang et al. reported a minimally invasive reduction technique for irreducible femoral neck fractures through the interaction between the head and stem of the femur [19, 20]. Here, we proposed a minimally invasive clamp reduction technique via the anterior approach for the treatment of irreducible intertrochanteric femoral fractures. In this article, we report the results from 115 patients treated at the Affiliated Hospital of Jining Medical University from January 2015 to January 2021.

## Clinical data

### General data

The inclusion criteria were as follows: ① closed femoral intertrochanteric fracture; ② age > 18 years; ③ treatment with closed reduction in the traction bed and internal fixation with intramedullary nailing; ④ closed reduction three times not meeting good repositioning criteria; [7]. ⑤ Follow-up period of at least 12 months. A total of 115 patients who met the inclusion criteria were enrolled in this study.

There were 48 males and 67 females with a mean age of 78.7 years old (45–100 years old). Fifty-one cases were left-sided, and 64 cases were right-sided. Patients were further divided by the type of injuries: fall (91 cases), traffic accidents (12 cases), smashing (6 cases), and high falling (6 cases). The duration from injury until surgery was 1–14 days (mean 3.9 days). All patients underwent pelvic orthotopic X-ray tablets and pelvic CT 3D reconstruction examination before surgery to determine their fracture AO type: 15 patients were 31-A1, 67 patients were 31-A2 and 33 patients were 31-A3. Ninety-nine patients had one or more other complications, including 89 patients with cardiovascular diseases, 71 patients with respiratory diseases, 61 patients with endocrine diseases, and 41 patients with neurological diseases.

### Surgical methods

The fractures were fixed by closed or limited open reduction.

Closed reduction was performed on the orthopedic traction bed in the following order: abduction, external rotation, adduction, internal rotation or external rotation. Then, a C-arm X-ray was used to evaluate the quality of the reduction (force line and displacement).

- Force line evaluation criteria [21]: The neck-shaft angle is normal or mildly valgus [22] in the orthotropic position (we suggest an angle ranging from 123–140°), and the angle of anteversion is < 20° in the lateral position;
- Displacement evaluation criteria [23]: orthotropic fracture displacement less than medial cortical thickness, lateral displacement less than anterior cortical thickness.

If none or only one of the force line criteria or the displacement criteria are met, it is considered a poor reduction [7]; if both of the force line criteria are met but only one of the displacements is met, then it is considered an acceptable reduction.

Poor or acceptable reductions were considered unsatisfactory and were classified as irreducible intertrochanteric femur fractures. All patients in this group were considered unsatisfactory after 3 closed resets.

#### Limited open reduction and fixation

The treatment method was selected according to the fracture line position and the fracture displacement mechanism.

**Fig. 1:**
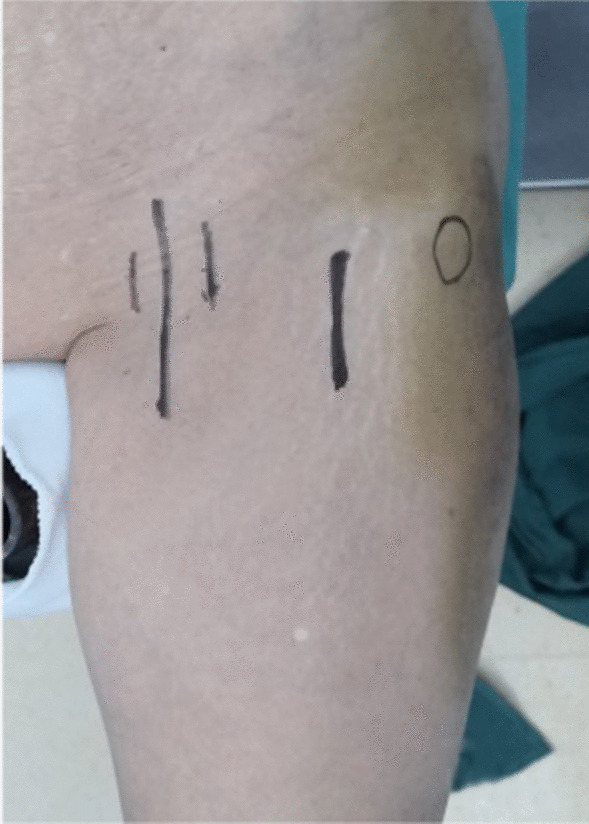
First, the femoral artery, femoral vein and femoral nerve are located in the inguinal area, and then a minimally invasive reduction incision is made approximately 2–3 cm below the inguinal area (femoral intertrochanteric line area)

**Fig. 2:**
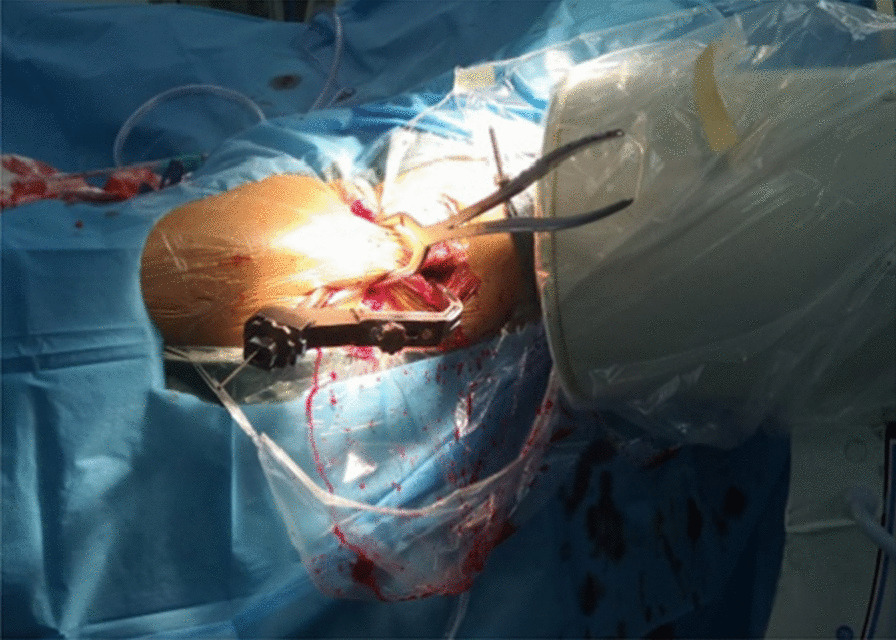
The reduction clamp with minimally invasive clamp reduction of the anterior approach does not interfere with the intramedullary nail implantation device

**Fig. 3:**
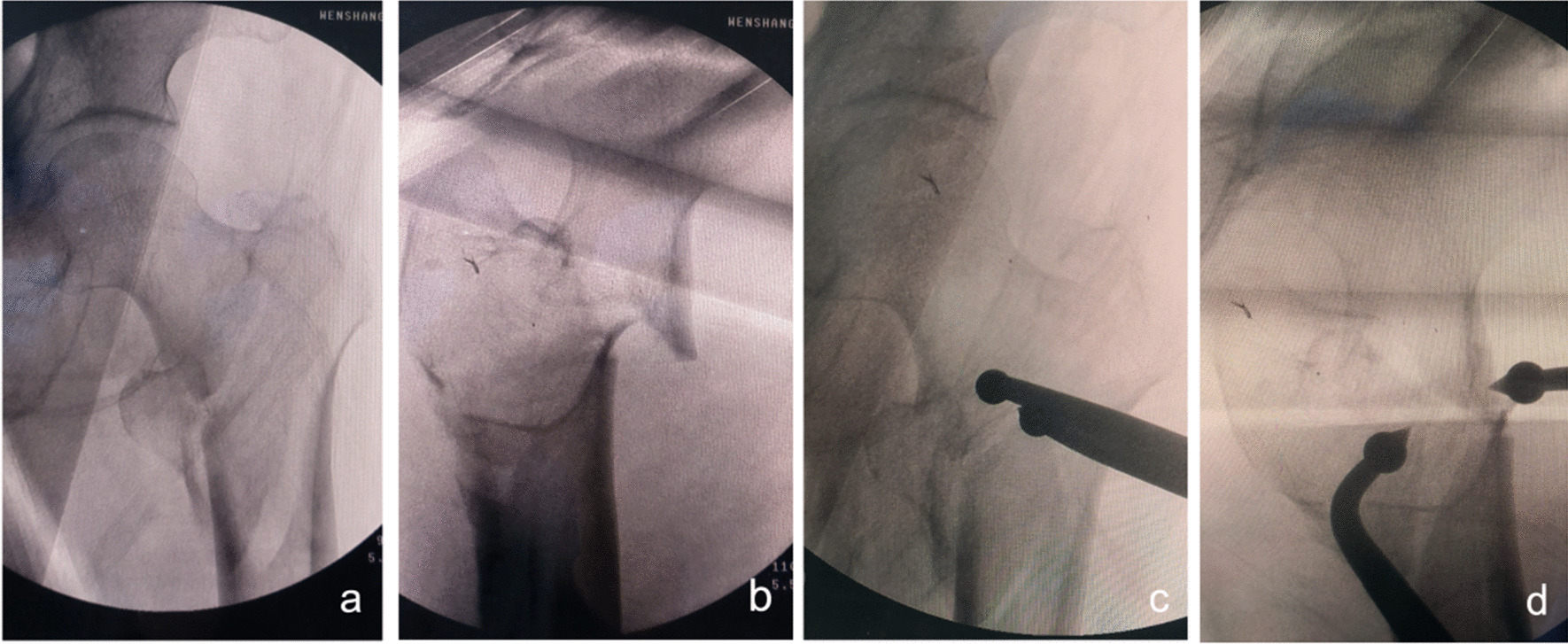
Anteroposterior and lateral X-ray films before and after reduction in patients with difficulty in reduction on the sagittal plane and pronation displacement of the proximal fracture segment **a**, **b** Before reduction; **c**, **d** After reduction (one head placed in front of the proximal fracture segment and the other head behind the greater trochanter)

**Fig. 4:**
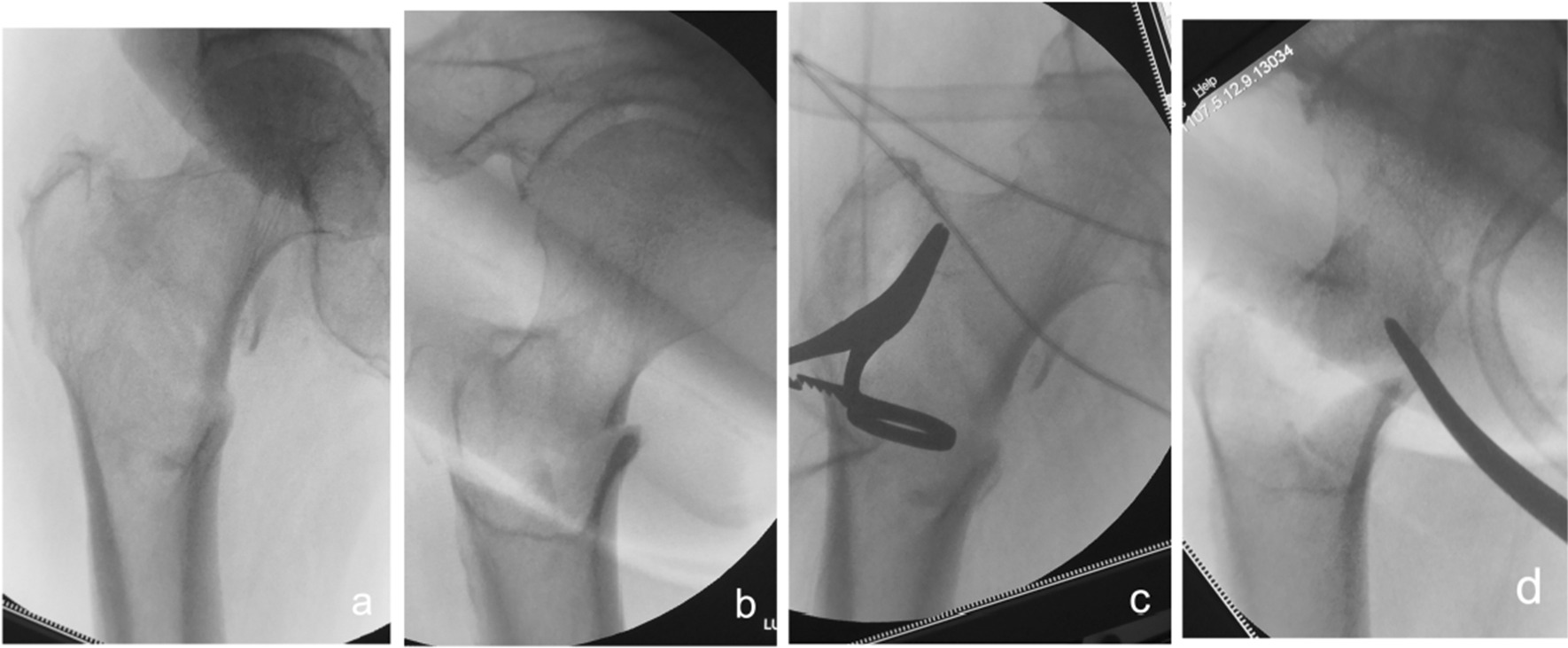
Anteroposterior and lateral X-ray films before and after reduction in patients with difficulty in reduction on the sagittal plane and supination displacement of the proximal fracture segment **a**, **b** Before reduction; **c**, **d** After reduction
② Problematic reduction in the coronal plane: (1) internal rotation displacement of the proximal fracture segment: In this group, three patients showed significant internal rotation displacement of the proximal fracture segment in the anteroposterior position and were basically normal in the lateral position. As shown in Fig. 5, a small incision is made in the intertrochanteric line area as described above. After reaching the surface of the bone, blunt separation is made obliquely against the bone surface toward the lesser trochanteric area to allow insertion of one tip of the pelvic reduction forceps into the gap. Then, a small cut was made lateral to the greater trochanter to insert the other tip of the pelvic reduction forceps. The fracture was repositioned with pelvic reduction forceps and fixed by an intramedullary nail after good reduction was fluoroscopically confirmed. (2) External rotation displacement of the proximal fracture segment: Four patients showed significant external rotation and coxa adducta of the proximal fracture segment on the anteroposterior X-ray and were generally normal on the lateral tablet. The proximal nail incision of the greater trochanter was enlarged, partially exposing the fracture line of the lateral wall of the greater trochanter, and the bone tenaculums clamped the mouth opening of the fracture line of the lateral wall, thus reducing the fracture (see Fig. 6). Perspective confirmed that the fracture was well restored, and then fixation was achieved by the intramedullary nail.

① Problematic reduction in the sagittal plane: (1) Pronation displacement of the proximal fracture segment: Among 21 patients, the fracture alignment was normal in the anteroposterior position; however, in the lateral position, the proximal fracture segment was clearly displaced anteriorly by rotation. The femoral artery, femoral nerve and femoral vein were located in the inguinal region, and the intertrochanteric line was located just outside and below these three important structures. As shown in Figs. 1 and 2, a longitudinal incision of approximately 3.0–4.0 cm was made in this area by blunt dissection to the bone surface using vascular forceps, where the proximal fracture segment was palpated with marked anterior rotation. In addition, a small incision, 1.0–1.5 cm, was made in the posterior trochanteric area behind or under the greater trochanter on the posterolateral aspect of the corresponding front incision and was separated to the bone surface or subfascia. The proximal fracture segment was reduced with pelvic reduction forceps through the two incisions (see Fig. 2), combined with distal fracture segment rotation and adjustment, and then fixed with an intramedullary nail after the anteroposterior and lateral fluoroscopic fractures had reached a good reduction (see Fig. 3). Sometimes, it is difficult to achieve anatomical reduction, but the positive and lateral positions can facilitate positive cortical contact rather than negative cortical contact to avoid fixation failure. The reduction of other types of irreducible intertrochanteric fractures described below also follows this criterion. (2) Supination displacement of the proximal fracture segment: Fourteen patients showed shortening of the femoral neck in the frontal view and supination displacement of the proximal fracture segment in the lateral view. The proximal fracture segment was inserted into the medullary cavity of the distal fracture segment due to postrotation and was obstructed by the anterior wall of the distal fracture segment, making it difficult to achieve a good reduction. To fix the issue, a small incision was made in the intertrochanteric area as described above. After blunt dissection, vascular forceps were inserted into the medullary cavity between the broken ends of the fracture, prying and repositioning, correcting the impaction (see Fig. 4), and sometimes further correction and maintenance by pelvic reduction forceps was needed. Fluoroscopy was utilized to confirm that the fracture was well repositioned and then fixed with an intramedullary nail.

**Fig. 5:**
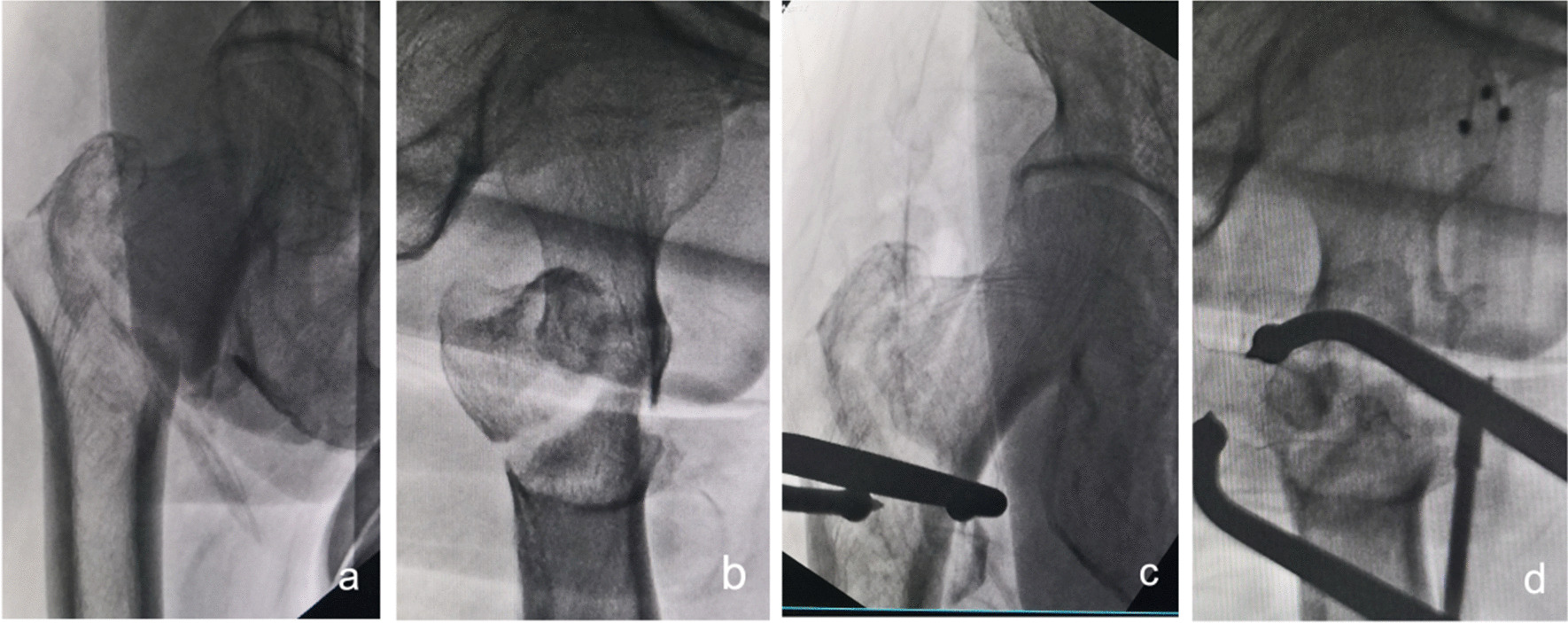
Anteroposterior and lateral X-ray films before and after reduction in patients with difficulty in reduction on the coronal plane and internal rotation displacement of the proximal fracture segment **a**, **b** Before reduction; **c**, **d** After reduction (one head placed on the inner side of the proximal fracture segment and the other head on the outer side of the greater trochanter)

**Fig. 6:**
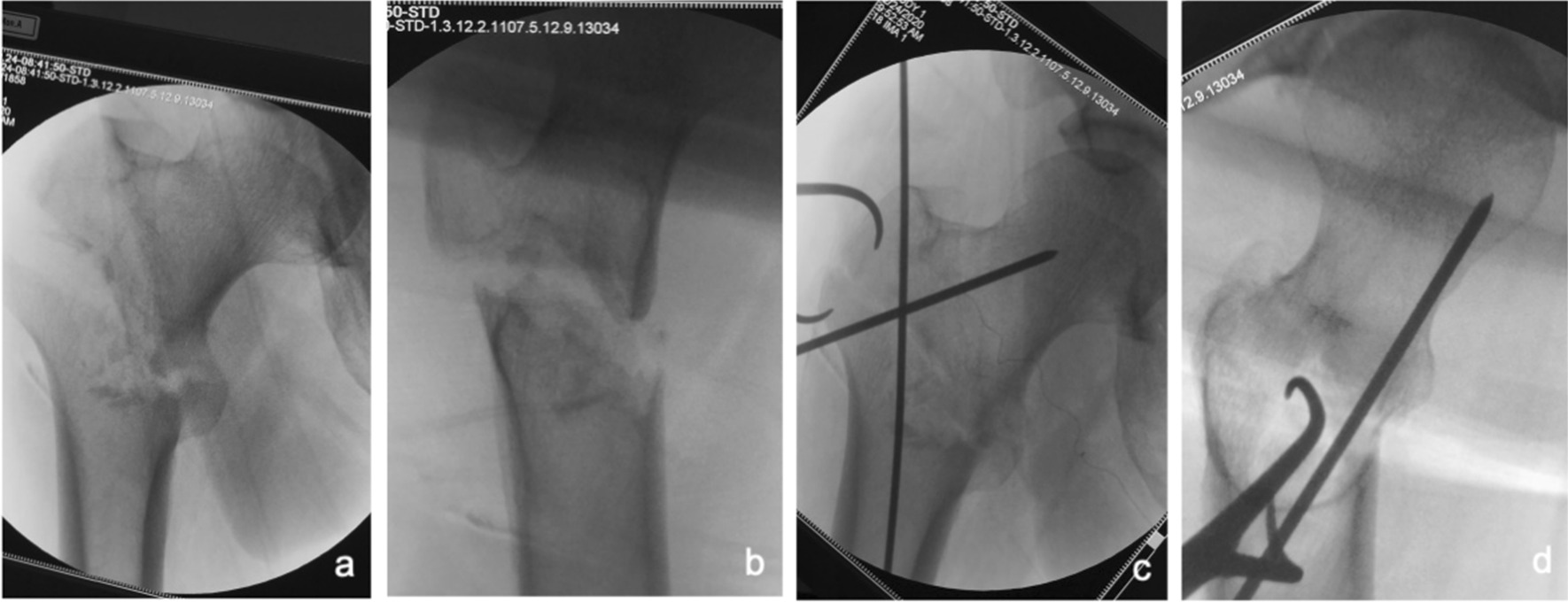
Anteroposterior and lateral X-ray films before and after reduction in patients with difficulty in reduction on the coronal plane and external rotation displacement of the proximal fracture segment **a**, **b** Before reduction; **c**, **d** After reduction
③ Problematic reduction in both sagittal and coronal planes: (1) Pronation and internal rotation displacement of the proximal fracture segment: The anteroposterior and lateral X-rays of 15 patients in this group showed that the proximal fracture segments presented pronation in the sagittal plane and internal rotation in the coronal plane. The reduction approach and reduction procedures are basically the same as in those with internal rotation shift in the proximal fracture segment of the coronal plane and with reduction difficulties. As both sagittal and coronal displacements need to be corrected, the reduction forceps are clamped at an angle between the sagittal and coronal sides to achieve good reduction in both planes (see Fig. 7). (2) Pronation and external rotation displacement of the proximal fracture segment: In four patients, in addition to restoring the pronation displacement through the anterior approach clamp, the bone tenaculums were applied simultaneously to the lateral wall fracture to correct the external rotation displacement, as shown in Fig. 8. (3) Supination and external rotation or internal rotation displacement of the proximal fracture segment: In two patients, the anterior approach hemostatic clamp was corrected for supination displacement by prying at the fracture end, and then the pelvic reduction clamp anterior approach was used to correct the internal rotation shift or the bone tenaculums to correct the external rotation displacement by clamping the lateral wall (see Fig. 9). Due to the presence of soft tissue hinges, good reduction was easily achieved with mild rotational adjustment of the distal fracture segment. After we confirmed that the fracture had achieved good reduction by fluoroscopy, the fracture was fixed with an intramedullary nail.

**Fig. 7:**
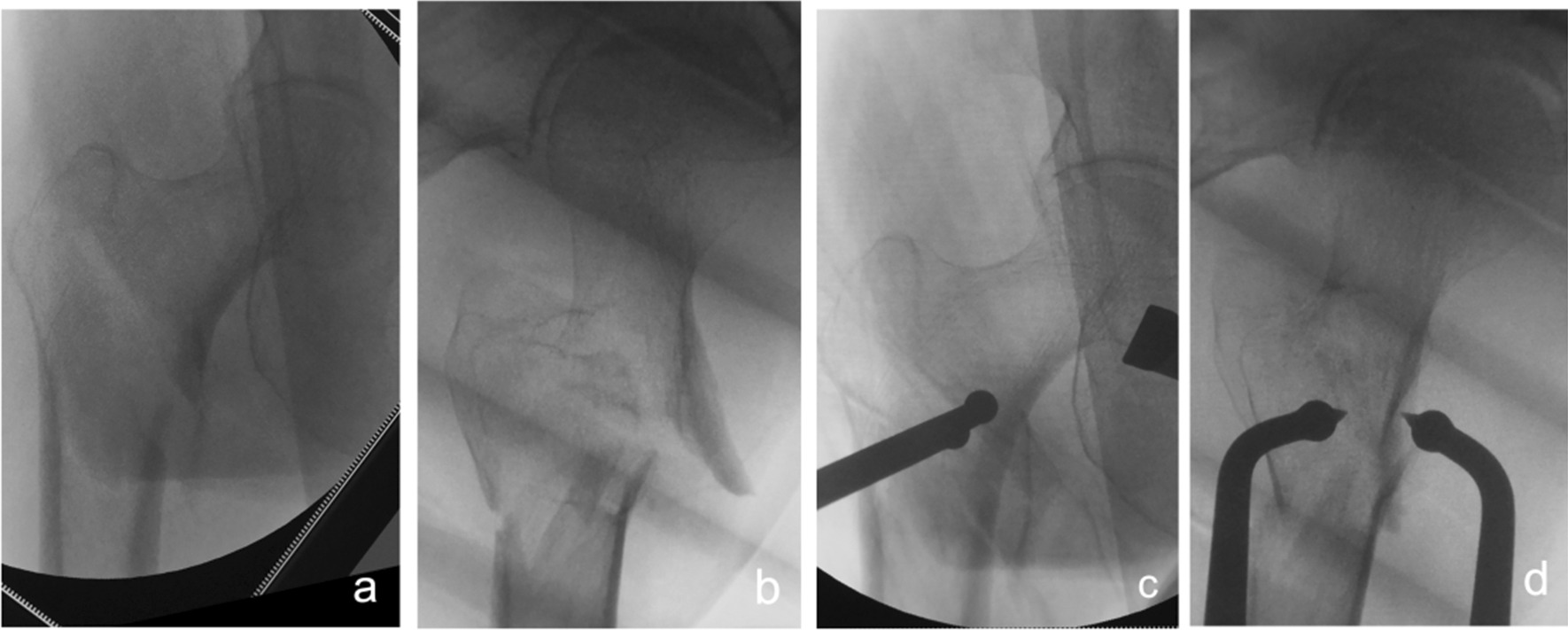
Anteroposterior and lateral X-ray films before and after reduction in patients with difficulty in reduction on both sagittal and coronal planes and pronation and medial rotation displacement of the proximal fracture segment **a**, **b** Before reduction; **c**, **d** After reduction (one head placed in front of the proximal fracture segment and the other head behind the greater trochanter)

**Fig. 8:**
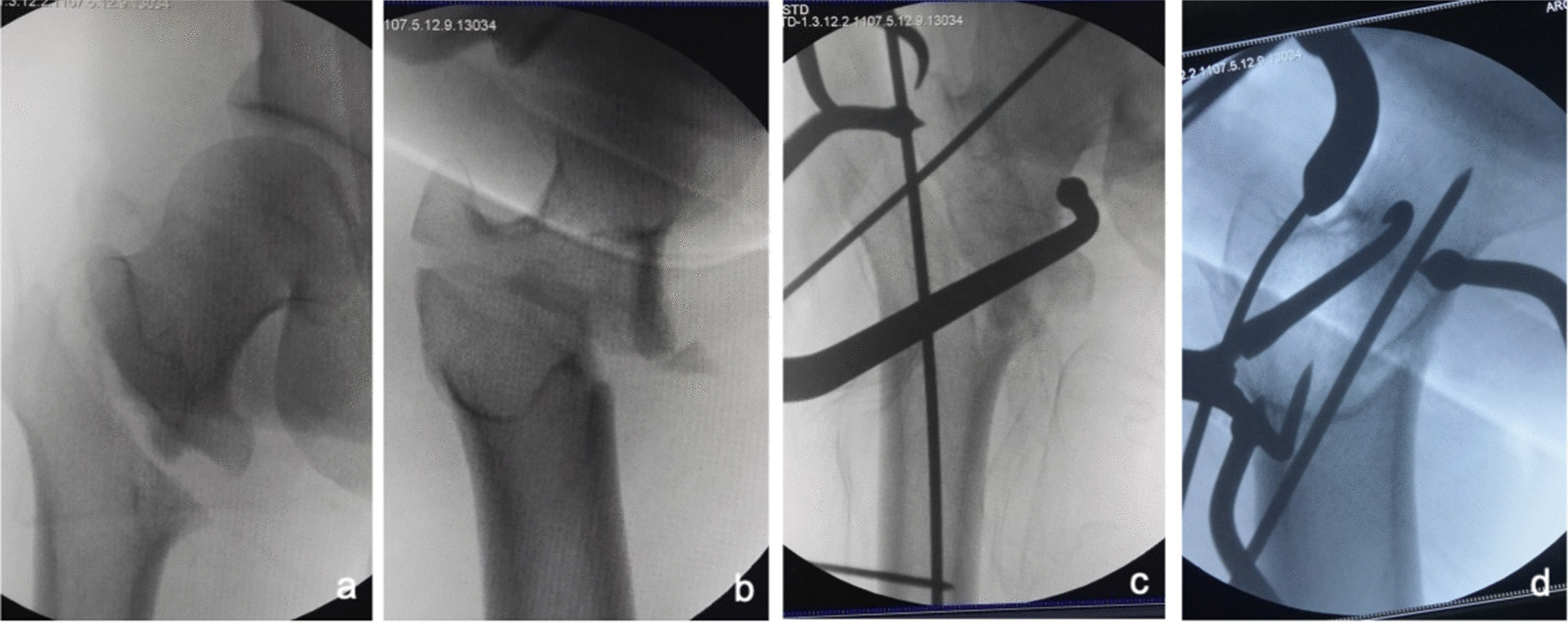
Anteroposterior and lateral X-ray films before and after reduction in patients with difficulty in reduction on both sagittal and coronal planes and pronation and external rotation displacement of the proximal fracture segment **a**, **b** Before reduction; **c**, **d** After reduction (one head placed in front of the proximal fracture segment and the other head behind the greater trochanter)

**Fig. 9:**
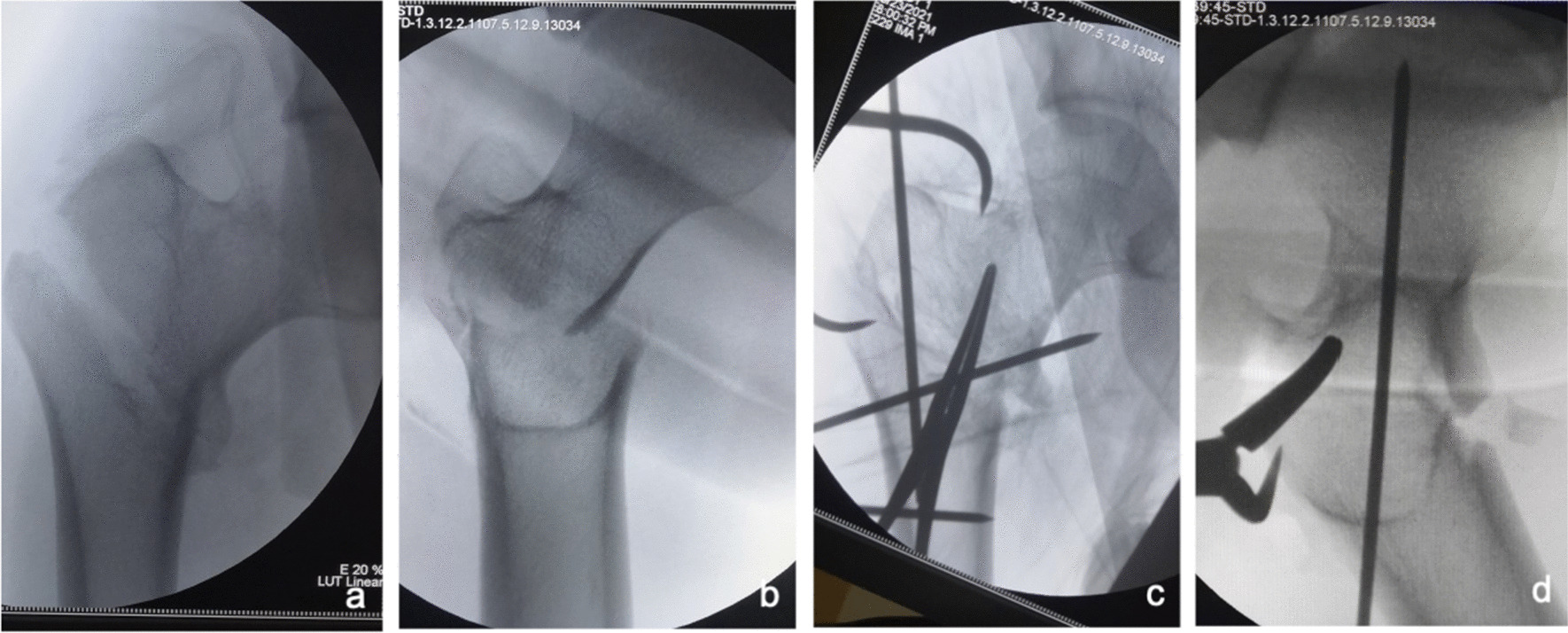
Anteroposterior and lateral X-ray films before and after reduction in patients with difficulty in reduction on both sagittal and coronal planes and supination and internal rotation or external rotation displacement of the proximal fracture segment **a**, **b** Before reduction; **c**, **d** After reduction
④ Problematic reduction related to the lateral wall: A total of 33 patients had reverse oblique intertrochanteric fractures. The anteroposterior view showed more obvious abduction on the lateral wall, while the lateral view primarily showed pronation after closed traction. As shown in Fig. 10, we made an incision 3.0–4.0 cm anterior to the tip of the lateral wall, which is slightly proximal to the lateral level of the femur, and then bluntly dissected to the femoral stem using reduction forceps to clamp the pronated, abducted lateral wall for the reduction. Once we confirmed that the fracture had achieved good reduction by fluoroscopy, the fracture was then fixed with an intramedullary nail.

**Fig. 10:**
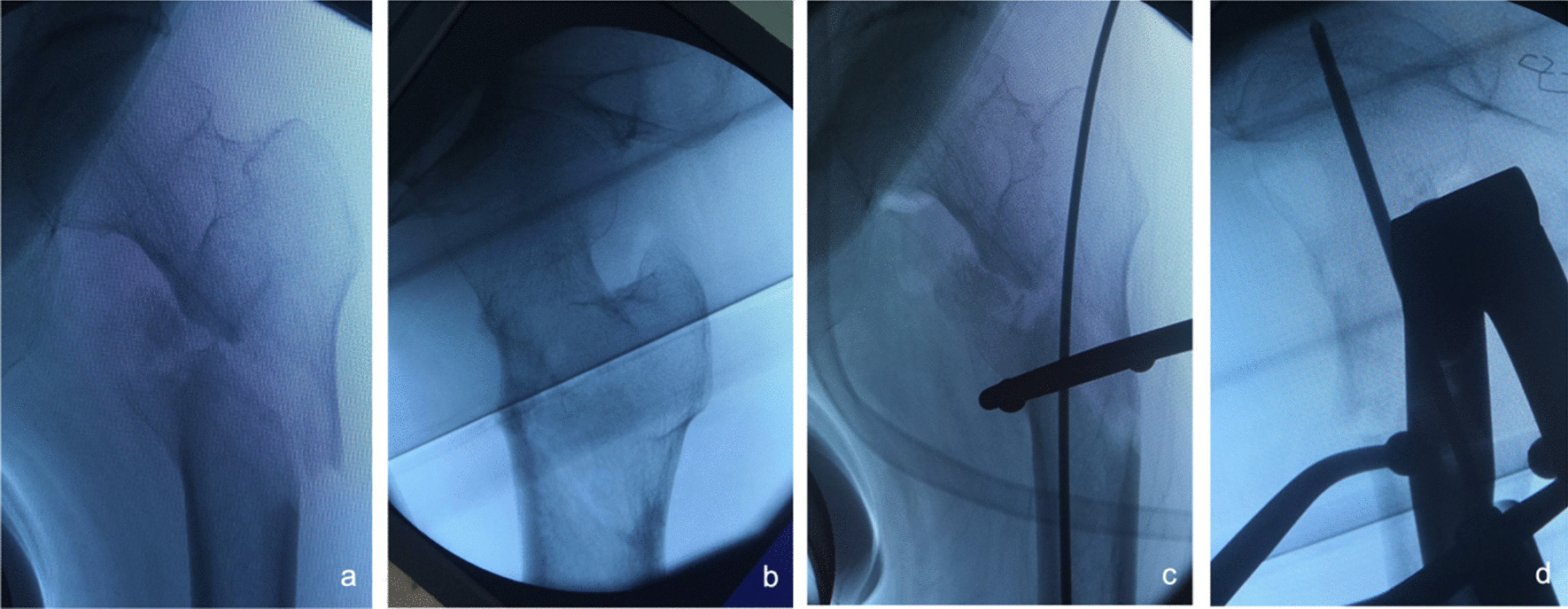
Anteroposterior and lateral X-ray films before and after reduction in patients with difficulty in reduction associated with the lateral wall **a**, **b** Before reduction; **c**, **d** After reduction
⑤ Problematic reduction associated with displacement of the proximal femoral fracture segment: In 19 patients, traction reduction was difficult because the fracture line occurred in all directions and the fracture segment was displaced in various directions. As shown in Fig. 11, for these patients, we chose to make an anterior incision slightly proximal to the level of the most obvious fracture displacement and then bluntly dissected to the femoral stem, which was reduced using a reduction forceps clamp. Then, we used the intramedullary nail to fix the fracture after fluoroscopy confirmed that the fracture was well set.

**Fig. 11:**
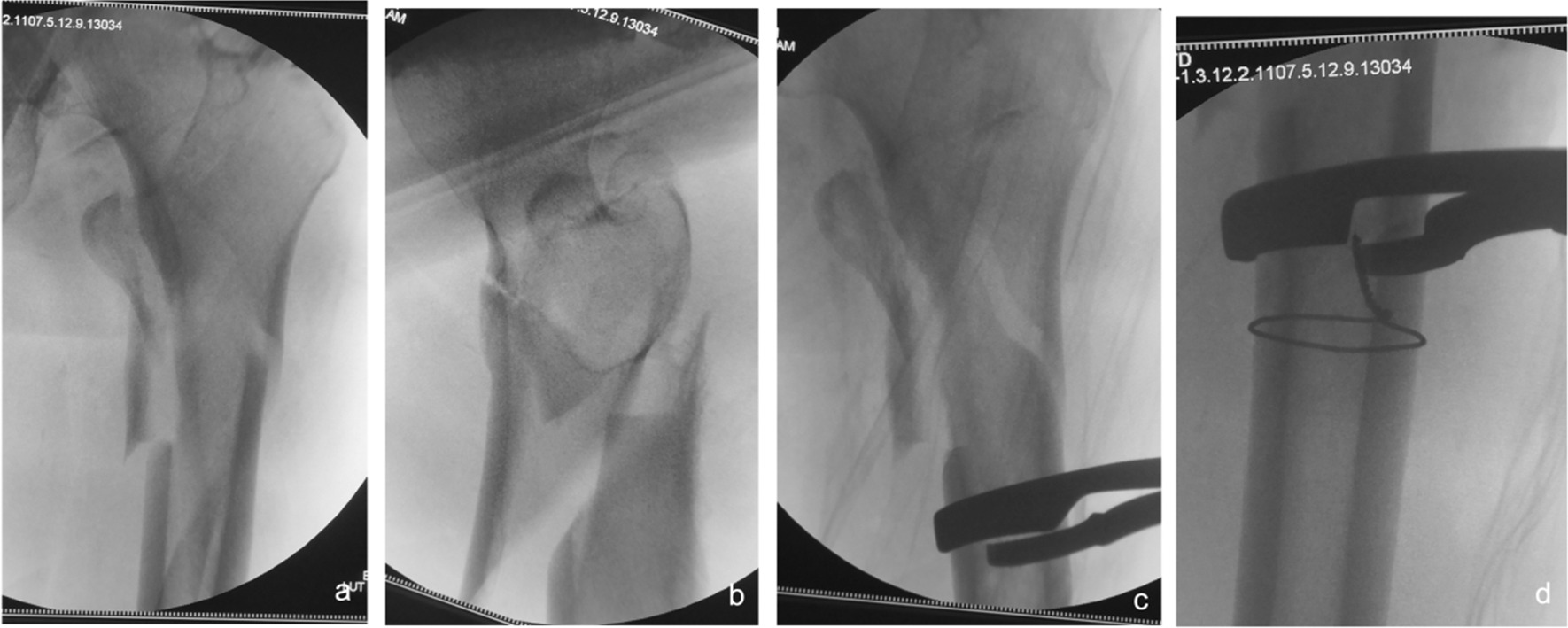
Anteroposterior and lateral X-ray films before and after reduction in patients with difficulty in reduction associated with proximal femoral fracture displacement **a**, **b** Before reduction; **c**, **d** After reduction

Caution: (1) Pelvic reduction forceps are of various sizes and we generally choose the large one since the hip muscles are relatively thick and sturdy. Only the large ones can easily grip the broken end of the fracture, and will not damage muscles and other tissues between the two heads of the forceps. (2) During the placement of the Pelvic reduction forceps, the bolt is generally taken off and two heads are completely separated. Place one head in the appropriate position of the proximal fracture segment under the guidance of operator's fingers, so as to avoid iatrogenic subsidiary injury. Then, partially close two heads, and place the other head on another pressing point. Clamp the two heads together until the fracture reduction can be achieved. Finally, screw on the bolt to maintain the reduction. (3) All of the above reduction requires the corresponding rotation adjustment of the distal fracture segment. At the same time, touch the broken end of the fracture with the fingers, so as to preliminarily judge the degree of reduction. Then, confirm the judgement through positive and lateral fluoroscopy.

### Postoperative management

Patients were not allowed to bear weight within 1.5 months after surgery, but they could perform passive or active hip flexion and extension exercises under protection; then, they could perform partial weight-bearing training under protection and start full weight-bearing when the fracture had healed under X-ray.

## Results

The time of fracture reduction, follow-up and fracture healing are shown in Table 1. All fractures that received limited incision achieved good reduction between 10 and 32 min (mean 18 min) post treatment. All patients were followed up for 12–27 months (mean of 17.9 months) after surgery. In two cases with anterior displacement of the proximal fracture segment by rotation, the one-month postoperative review revealed the withdrawal of the spiral blade, fracture displacement, and failure of internal fixation due to premature unprotected weight-bearing. The patients were advised to stay in bed strictly and not to bear weight. One patient died 1 year after surgery due to skin infection caused by the tail of the spiral blade, which resulted in infectious shock and hypostatic pneumonia. Another patient died of hypostatic pneumonia due to long-standing bed rest. In one patient, pronation displacement occurred in the proximal fracture segment, and comminuted fractures occurred in both the greater and lesser trochanter. Four months after surgery, the spiral blade was cut out, the fracture did not heal, and the internal fixation failed. Finally, the half-hip joint was replaced to restore walking function. Six patients with reverse oblique intertrochanteric fractures had pronation and abduction displacement again in the lateral wall after internal fixation; these patients were advised to bear weight later to protect the affected limb from trauma, and all of them achieved bony healing at the regular outpatient reviews. The rest of the patients who had successful fracture reduction all achieved bony healing. The healing time ranged from 3 to 9 months (mean of 5.7). While two patients died and one patient had a spiral blade cut-out half-hip replacement, 91 of the remaining patients had an excellent Harris score of hip function at the last follow-up, and 21 patients had a good Harris score.

**Table 1:** Time for surgical reduction, follow-up and bony healing

Description types	Time	Average value
Time for fracture reduction	10–32 min	18 min
Follow-up time	12–27 month	17.9 month
Bony healing time*	3–9 month	5.7 month

*Excluding 3 patients, 2 of them internal fixation failed and died; 1 joint replacement after internal fixation failure

## Discussion

### Rationale of the minimally invasive clamp reduction technique

**Fig. 12:**
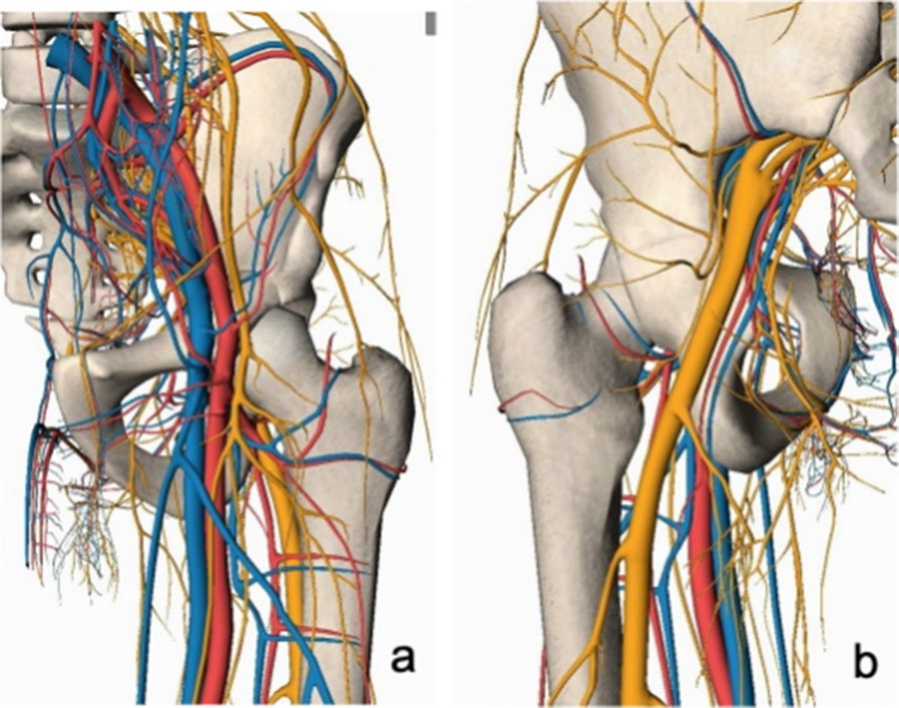
Important vascular, nerve, and other structures were absent in the anterior, posterior, and lateral regions of the femoral trochanteric region **a** anterior; **b** posterior

Irreducible intertrochanteric femoral fractures tend to malunion and severely affect the function of the hip. Good reduction can improve the quality of fixation, reduce the probability of internal fixation failure [24] and facilitate early functional exercise [25, 26]. The presence of the femoral artery, femoral vein, femoral nerve and sciatic nerve in the hip makes resetting difficult, and the risk of medically induced vascular and nerve injury is significantly increased. However, none of those critical vascular or nervous structures are located in the anterior, lateral and posterior sides of the femoral trochanteric region (see Fig. 12); therefore, we chose two entrances of the clamp reduction based on this anatomical feature. First, blunt dissection was performed anteriorly to the intertrochanteric line area, away from the femoral artery, femoral vein and femoral nerve, and only part of the rectus femoris, sartorius and vaginoplasties were damaged. These minor injuries were negligible because of longitudinal dissociation. The sciatic nerve is located near the midpoint of the line between the ischial tuberosity and the femoral greater trochanter. We chose to make a small incision posterior to the greater trochanter, with a depth of incision not exceeding the deep fascia, to allow access to one tip of the pelvic reduction forceps. Due to fascial restrictions, the reduction forceps can only grip the posterior aspect of the greater trochanter during reduction and cannot move around. This ensures that the sciatic nerve is not damaged. It is important to note that during blunt dissection of the lesser trochanteric region, by entering bluntly from directly anterior to the intertrochanteric line of the femur and obliquely dissecting to the lesser trochanter region after reaching the bone surface, even if the surgical location is close to the abovementioned important vascular and neural structures, damage to those structures can be avoided. The same principle also applies to the subtrochanteric area, where there were no important vascular or nerve structures around the femur, especially anteriorly, and it was safe to make a blunt dissection from the anterior side to the bone surface.

The key point of the minimally invasive technique of head-stem interaction for irreducible femoral neck fracture is controlling the femoral head by the Kirschner wire inserted in the anterior or anterolateral side, which moves the femoral head to achieve anatomical reduction [19, 20]. Inspired by this technique, we have proposed minimally invasive access from the anterior side of the intertrochanteric region of the femur to safely reach the intertrochanteric region, the lesser trochanteric region, and the lateral wall region of the greater trochanter. In addition, we controlled the movement of the proximal fracture segment using a reduction clamp to correct the abnormal displacement in all directions. Therefore, we believe that the "*safe gate*" for minimally invasive reduction of irreducible intertrochanteric fractures is directly anterior to the intertrochanteric line.

### Management of different types of fractures with difficult reduction

Tong et al. [27] classified irreducible intertrochanteric femoral fractures into three types: sagittal, coronal, sagittal and coronal, plus two types associated with the lesser and greater trochanter. There are a total of five types of irreducible intertrochanteric femoral fractures based on the fracture line alignment. Most of these fractures require a limited incision to assist in reduction, and some experts think that good fracture reduction can be achieved without an additional incision by enlarging the spiral blade incision or the proximal entry of the greater trochanter [11].

Our treatment demonstrated the following.

1. Problematic reduction in the sagittal plane: For the pronated displacement of the proximal fracture segment, the above approach [11] is acceptable, but it causes more damage as it requires dissection close to the periosteum, and further dissection is required to determine reduction. In addition, for patients with supine displacement of the proximal fracture segment and insertion into the medullary cavity of the distal fracture segment to form bony strangulation, this approach is more difficult and more traumatic to separate than the anterior approach, which is simpler and less traumatic to pry and reposition.
2. Problematic reduction in the coronal plane: For a significant medial displacement of the proximal fracture segment, reduction through a spiral blade incision is more difficult, which may cause a larger degree of soft tissue damage and an increased risk of injury to the femoral artery, vein and femoral nerve. In contrast, with the anteriorly minimally invasive clamping technique, the anteromedial soft tissues only need to be separated to the center of effort at the medial wall of the proximal fracture segment, avoiding extensive stripping of soft tissue in the area anterior to the intertrochanteric line while reducing trauma.
3. Problematic reduction in both sagittal and coronal plane reductions: When reduction occurs through a spiral blade incision, this type of fracture has the difficulties and disadvantages of the two types mentioned above, whereas a good reduction can be achieved by adjusting the angle of the clamp in a minimally invasive manner with a trans-anterior approach.
4. Problematic reduction of the lateral wall: If the fracture line at the most distal part of the lateral wall is near the spiral blade incision, exposure through this incision is possible, but it is also difficult to maintain the reduction. If the fracture line is distant from the spiral blade incision, exposure in only one incision will further increase the trauma. Therefore, an anterior approach with clamping reduction can be chosen, which is not only less traumatic but also facilitates the maintenance of the fracture. Difficulty in reduction associated with displacement of the proximal femoral fracture segment is managed similarly to that related to the reduction of the lateral wall.

### Advantages and cautions for using minimally invasive clamping and reduction techniques

Advantages: ① We only performed a simple blunt dissection at the front of the hip, as long as the displaced fracture end is palpated by the fingers ("*Only Touch Not See*"), and then performed clamp reduction. Although small incisions are made more anteriorly and posteriorly or laterally in the hip and the skin integrity is disrupted, there is minimal damage to the soft tissues surrounding the broken end of the fracture, achieving true minimal invasion. During follow-up, no patient had complaints of soft tissue discomfort near the anterior-assisted reduction of the hip incision ② Traditional reduction methods have more difficulty maintaining reduction, and the risk of redisplacement is high. Kirschner wire maintained reduction is one of the commonly used methods, but it sometimes interferes with subsequent intramedullary nail implantation. If the Kirschner wire breaks during the reaming process, it is difficult to remove, which will greatly increase the operation time and the risk of infection. The presence of a bolt in the reduction clamp facilitates maintenance of the reduction, thus avoiding manual maintenance, which can easily lead to redisplacement [10, 17]. Good maintenance of the fracture also helps to avoid repeated intraoperative fluoroscopy and reduces radiation damage [10, 17, 18]. In addition, the reduction forceps do not interfere with subsequent intramedullary nailing fixation. The reduction forceps used to maintain the fracture are placed anteriorly (difficulty in coronal reduction), anterolaterally (difficulty in sagittal and coronal reduction) or laterally (difficulty in sagittal reduction) to the hip, whereas the femoral neck is mostly anteriorly inclined and the intramedullary nailing mounting instruments are placed on the posterolateral side of the thigh, without interfering with the reduction forceps (see Fig. 2).

**Fig. 13:**
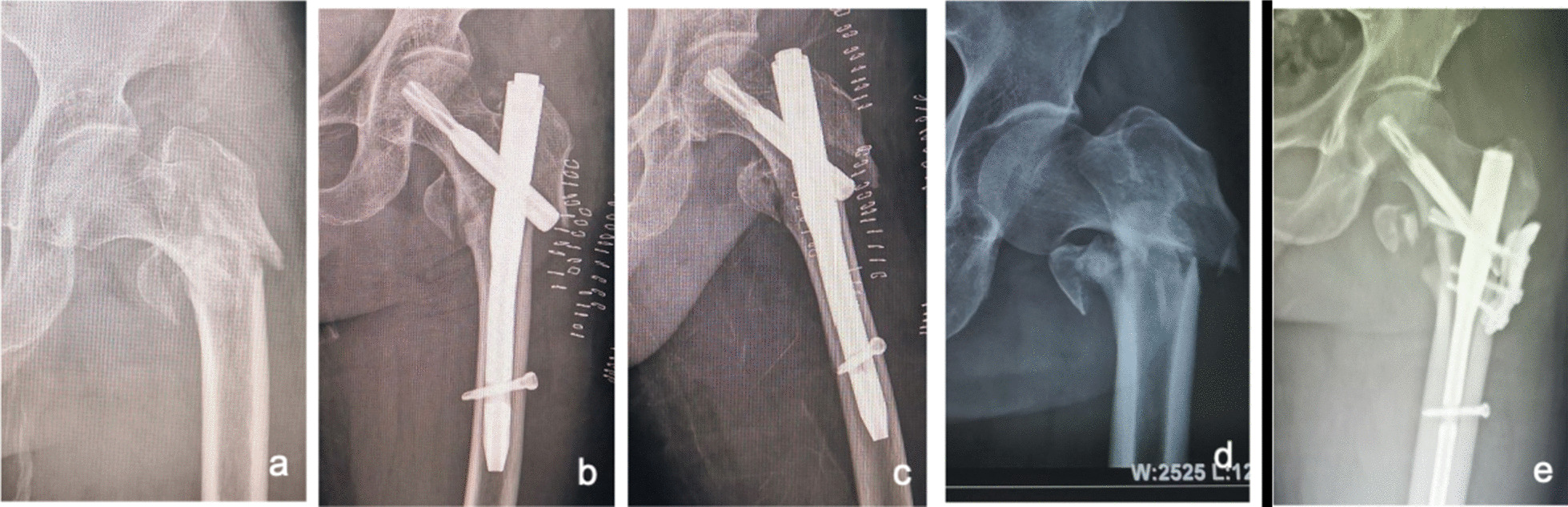
After the fixation of intramedullary nails in irreducible intertrochanteric femoral fractures associated with the lateral wall (mostly reverse oblique intertrochanteric fractures), the lateral wall is easy to displace again (**a**–**c**); lateral wall reinforcement can avoid redisplacement and improve fracture stability (**d**, **e**)

Cautions: ① Strong hip limb muscles can lead to deformity and displacement of the fracture, which is sometimes problematic for bone tenaculums to counter. The large working distance of the pelvic resetting forceps facilitates counteracting the powerful perihip muscles. In addition, the blunt tip of the pelvic reduction forceps is less likely to cause collateral damage. For this reason, we routinely prepare pelvic reduction forceps or fracture clamping forceps preoperatively, and when traction reduction is difficult, an anterior approach with limited incision clamping is preferred for resurfacing and maintaining reduction, followed by intramedullary nailing. ② Irreducible intertrochanteric femoral fractures associated with the lateral wall (mostly reverse oblique intertrochanteric fractures) have a high rate of internal fixation failure [28, 29]. Even with good intraoperative clamping and reduction, sometimes the spiral blade of the intramedullary nail does not provide effective fixation of the lateral wall, and postoperative redisplacement of the lateral wall occurs (see Fig. 13a–c), so it is recommended that the lateral wall is reinforced at the same time as the intramedullary nail fixation (see Fig. 13d, e) to reduce the rate of fixation failure [29]. ③ The rate of difficult fractures at our center was 32.76% (115/351), which is higher than the previously reported rate of irreducible fractures in all intertrochanteric fractures (3–17%) [2, 4, 6]. In the analysis of the above literature reports, only AO/OTA types 31-A1 and 31-A2 were included, without including type 31-A3. However, most of the actual 31-A3 types are irreducible fractures [7]. In addition, the partial data of our group came from consultation cases in many hospitals due to difficulties in fracture treatment, so the proportion of irreducible intertrochanteric fractures was large, leading to a data bias. The high proportion of irreducible femoral intertrochanteric fractures suggests the importance of mastering the minimally invasive clamp reduction technique. ④ In extremely osteoporotic geriatric femurs, lateral wall blow-out fractures can occur while implanting nails. We can strengthen the lateral wall with the plate (Fig. 13d, e), application of circumferential cerclage cable [29], and suture with nonabsorbable suture, etc. ⑤ When determining the nail entry portal, it should be slightly more medial than the conventional site on the greater trochanter because due to the pushing effect of soft tissue, it is easy to eventually lead to the outer opening. The consequence of the outer opening easily leads to different degrees of hip varus and left lameness.

In conclusion, for irreducible intertrochanteric femoral fractures, limited incision through the anterior approach and clamping with pelvic reduction forceps or fracture champing forceps can achieve minimally invasive reduction, reducing the difficulty of reduction and the degree of trauma and improving the fixation effect. However, this study only reported limited and single-center retrospective cases, and more multicenter prospective studies and controlled studies on the traditional incisional repositioning technique are needed to further clarify the efficacy.

## Data Availability

All datasets analyzed during this study are available from the corresponding author upon reasonable request.
